# Incidence, persistence and risk factors of fear of falling in older adults: cohort study (2008–2013) in Rio de Janeiro, Brazil

**DOI:** 10.11606/S1518-8787.2020054001939

**Published:** 2020-06-05

**Authors:** Flávia Moura Malini Drummond, Roberto Alves Lourenço, Claudia de Souza Lopes

**Affiliations:** I Universidade do Estado do Rio de Janeiro Instituto de Medicina Social Rio de JaneiroRJ Brasil Universidade do Estado do Rio de Janeiro. Instituto de Medicina Social. Rio de Janeiro, RJ, Brasil; II Universidade do Estado do Rio de Janeiro Faculdade de Ciências Médicas Rio de JaneiroRJ Brasil Universidade do Estado do Rio de Janeiro. Faculdade de Ciências Médicas. Rio de Janeiro, RJ, Brasil

**Keywords:** Aged, Fear, classification, Accidental Falls, Risk Factors, Cohort Studies

## Abstract

**OBJECTIVE:**

To evaluate the incidence and persistence of fear of falling in older adults and the clinical/functional, psychosocial and lifestyle-related risk factors.

**METHODS:**

A longitudinal study with 393 community-dwelling older adults aged 65 years and over (110 men/ 283 women) resident in the North Zone of the city of Rio de Janeiro, Brazil. The fear of falling was assessed by the Falls Efficacy Scale-I-BR. The explanatory variables assessed were: number of comorbidities and medicines, history of falls, fracture from falling, use of walking aids, functional dependence in basic and instrumental activities of daily living, hearing and visual impairment, hand grip strength, walking speed, self-rated health, body mass index, depressive symptoms, cognitive impairment, living alone and activity level. Incidence, persistence and risk factors were estimated. Multivariate analysis was performed using Poisson Regression, obtaining relative risks (RR) and corresponding to 95% confidence intervals.

**RESULTS:**

Among the 393 participants, fear of falling occurred in 33.5% and was persistent in 71.3%. Incidence was found to associate with using seven or more medicines and reporting worse activity level than the prior year. Risk factors for persistent fear were: using seven or more medicines, a history of one or two falls, reduced walking speed, hearing impairment, cognitive impairment, depressive symptoms and poor or very poor self-rated health.

**CONCLUSION:**

Fear of falling is a frequent and persistent condition. Many factors related to persistent fear showed no association with the incidence of fear, emphasizing the need for focused strategies to reduce risk factors that may be associated with the chronification of fear of falling.

## INTRODUCTION

Fear of falling (FOF) can be defined as a constant worry about falling, which limits activities of daily living^1^. It can be assessed by the concept of self-efficacy, which evaluates the individual’s confidence in performing activities without falling; being the most used way in studies to assess FOF^2^. Self-efficacy assessment assesses the individual’s concern with performing activities of daily living without falling, which assesses the cognitive part of fear of falling^1^. For older adults, the adverse consequences of this fear include: greater risk of falling, functional constraints, lesser quality of life, lower level of physical activity and even higher mortality^3^.

The prevalence of FOF is high, ranging from 20.8% to 85%, according to a systematic review^6^. This amplitude results mainly from differences in the assessment instrument and study sample characteristics.

Studies show that the main risk factors for incidence are: being a woman, more advanced age, having a history of falls, impaired gait or balance and depression or depressive symptoms. Incidence rates range from 13% to 45%^7^. However, these studies are also rather heterogeneous in their populations, particularly regarding age ranges and sex (some studies were conducted only among women)^7,8^.

Few studies have evaluated the persistence of fear of falling, which ranged from 46% to 80%. With different risk factors when compared with occurrence^7,9^. The main factors involved were: history of falls, living alone, cognitive impairment, body mass index, use of medicine for anxiety or depression and gait disturbance^7^.

Differentiating risk factors for incident and persistent fear is essential to establish appropriate assessment and intervention strategies. Another important point is that most longitudinal studies that evaluate fear of falling occurrence and related risk factors are based on one simple question^7^Only one study has assessed fear of falling using a structured scale, the Falls Efficacy Scale (FES). However, this study was conducted among older adults admitted to a care center, who were evaluated only just before discharge^15^. Another important issue is that our study is the first one conducted in Brazil and Latin American to assess fear of falling in a longitudinal design, which can provide answers regarding incidence, persistence and risk factors to be compared with international studies and understand this condition that affect quality of life in older adults.

To the best of our knowledge, our study is the first to use a structured scale to investigate fear of falling in older adults in the community, seeking to assess the occurrence and persistence of such a fear and related clinical/functional, psychosocial and lifestyle risk factors, at a four-year follow-up (2008-2013).

## METHODS

### Study Design, Context and Sampling

Baseline and follow-up data were drawn based on the Rio de Janeiro section of the study of Frailty in Brazilian Older Adults^16^. The study population comprised older-adult clients of a health insurance operator. The inclusion criteria were: having been a client of the operator for at least 12 months; being 65 years old or more and living in one of the neighbourhoods of the administrative North Zone of the municipality of Rio de Janeiro. The exclusion criteria were: presenting an active neurological or psychiatric disorder, mini-mental state examination (MMSE) score of less than 14 and severe hearing and visual impairments that would hinder questionnaire answering.

The sample was selected by inverse random sampling among the individuals on the health insurance operator’s records and stratified according to sex and age groups (65–74, 75–84, 85–94, and ≥ 95 years). We used a census instead of sampling the strata of men and women from 95 to 99 years of age or 100 or more years of age. The sample was estimated based on the prevalence of frailty syndrome, estimated at 12%. The prevalence of fear of falling is higher, allowing us to infer the incidence of fear of falling in our study.

At the 2009 baseline, 742 older adults were interviewed. Four years later, 86 (10.8%) had died and 70 (9.4%) had to be excluded due to cognitive impairment, sensory deficit or no longer walking. Then, 586 older adults remained eligible for follow-up, 32.9% (N = 193) of whom were lost. The losses included 135 older adults (23%), who refused to participate in the follow-up stage and 58 (9.9%), who were not localized after several contact attempts. Our study thus included 393 older adults (Figure).

**Figure:**
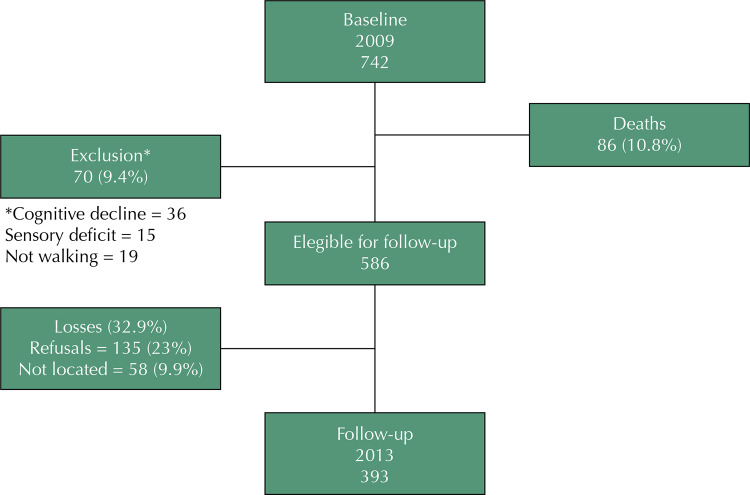
Flow diagram of study participants.

### Measures

#### Fear of falling

Fear of falling (FOF) was assessed using the Falls Efficacy Scale-International, in the version for use in Brazil (FES-I-BR), which is designed to evaluate self-rated self-efficacy concerning falling. This scale has 16 questions regarding the concern with falling when performing both domestic and social activities. The answers are: “not worried at all,” “a little worried,” “very worried,” and “extremely worried” about falling when performing such activities. The score ranges from 16 to 64 points. This variable was transformed into a dichotomous variable, in which 23 or more points correspond to being afraid of falling. This cut-off was chosen due to its association with an actual risk of falling^17,18^.

#### Clinical and functional variables

The information was collected and the variables categorized as follows: “number of medicines regularly used” (0-3; 4-6; ≥7), “number of comorbidities reported” (0-1; 2-3; ≥ 4), “history of falls in the prior year” (0; 1-2; ≥3), “fractured from falling” (No/Yes), “use of walking aids” (No/Yes), “self-reported hearing and visual impairment” (No/Yes).

The Katz and Lawton scales were used, respectively, to evaluate levels of independence in basic (BADL) and instrumental (IADL) activities of daily living^19,20^. Individuals who needed help or who did not perform at least one of the activities evaluated were considered dependent.

To evaluate muscular strength, hand grip strength was measured using a dynamometer, by measuring force kilogram (JAMAR Model J00105), applying cut-off points to consider sex and body mass index (BMI). Individuals in the first quintile were regarded as having reduced strength^21^. Walking speed was evaluated using a chronometer to measure the time needed to cover 4.6 meters, and then adjusted for height and sex. Individuals in the first quintile were considered to have diminished walking speed^21^. Walking speed was measured as follow: 4.6 meters divided by time in seconds (m/s).

Self-rated health was assessed by the following question and respective answer options: “Generally speaking, would you say that your health is: very good, good, fair, poor, or very poor?.” These answer options were subsequently grouped into three categories: “very good/good,” “fair” and “poor/very poor.”

Body mass index (BMI) was obtained by anthropometric measurements (weight in kg divided by height squared in meters). Based on these measurements, the older adults were categorized as: “undernourished” (BMI < 22), “eutrophic” (BMI from 22 to 27), and “overweight and obese” (BMI > 27).

#### Psychosocial variables

The existence of cognitive impairment was confirmed by the Mini-Mental State Examination (MMSE), considering 18/19 as the cut-off point for illiterate individuals and 24/25 for individuals with one or more years of schooling^22,23^. The existence of depressive symptoms was determined using the Geriatric Depression Scale (GDS), with cut-off at six or more points^24^. A simple “Yes/No” question was used to determinate if the individual lived alone.

#### Lifestyle Variable

Overall activity level was assessed by the question: “*Compared with a year ago, how would you describe your activity level?*” The answer options were: “better,” “the same,” or “worse.” This variable was transformed into the dichotomous variable “better/the same” and “worse.”

## Covariables

The covariables evaluated were: sex, age, schooling, marital status and income in minimum wages. These variables were included in the analysis as confounding variables in the adjusted models.

## Data Analysis

First, losses at the follow-up were examined for differentials between the losses and the older adults who participated in follow-up (2008-2013). Bivariate analyses were performed using chi-square (x^2^) or Fisher’s exact tests.

To analyze the risk factors for the incidence and persistence of fear of falling, the older adults were divided into two groups: (1) those who had no FOF at the baseline and had developed it at the follow-up, to evaluate the factors associated with the incidence; and (2) those who had FOF at the baseline and still have it at the follow-up, to evaluate factors associated with the persistence. Incident FOF was estimated by dividing the number of people who developed FOF at follow-up by the number of people who reported no FOF at any stage. Persistent fear was estimated by dividing the number of people reporting FOF in both phases by the number of people that reported no FOF.

Associations between the occurrence and persistence of FOF and the risk factors were assessed using Poisson Regression, considering over-dispersion using robust variance. Relative Risks (RR) and respective 95% confidence intervals (95%CI) were estimated.

Socioeconomic and demographic covariables that returned p-value < 0.10 in the bivariate analyses were included in the models as confounding variables. Then, considering these criteria, models were constructed separately for incidence and persistence. For incidence, two adjusted models were constructed: model 1 included sex and age, and model 2 included sex, age and all independent variables. For persistence, the models included two fitted models: Model 1 with sex, age and marital status, and Model 2 with sex, age, marital status and all the independent variables.

The statistical analyses were performed using SPSS for Windows, version 19.

The FIBRA-RJ study was authorised by the research ethics committee of the Hospital Universitário Pedro Ernesto (1850-CEP/HUPE). All participating individuals signed an informed consent form.

## RESULTS

Of the 586 eligible older adults who participated in the first phase of the study, 393 were examined at follow-up, resulting in a 67% response rate. No statistically significant differences regarding age, sex, schooling, marital status, income, dependence in ADL or existence of FOF at baseline were found between the older adults lost at the follow-up and the participants (Table 1).

**Table 1:** Socioeconomic and demographic characteristics of losses compared with those of participants in follow-up (2009-2013), Rio de Janeiro, RJ, Brazil.

	Eligible for follow-up (N = 586) n (%)	Losses and refusals (N = 193) n (%)	Final sample (N = 393) n (%)	p
Sex				0.248
Male	170 (29.0)	60 (31.1)	110 (28.0)	
Female	416 (71.0)	133 (68.9)	283 (72.0)	
Age				0.358
65–74 years	268 (45.8)	83 (43.0)	185 (47.1)	
75–84 years	248 (42.3)	82 (42.5)	166 (42.2)	
≥ 85 years	70 (11.9)	28 (14.5)	42 (10.7)	
Marital status				0.761
Married or with companion	259 (44.2)	86 (44.5)	173 (44.0)	
Divorced/separated	46 (7.8)	12 (6.3)	34 (8.7)	
Single	68 (11.6)	22 (11.4)	46 (11.7)	
Widowed	213 (36.4)	73 (37.8)	140 (35.6)	
Schooling				0.525
Illiterate	10 (1.7)	5 (2.5)	5 (1.2)	
1–5 years	125 (21.3)	45 (23.3)	80 (20.4)	
6–11 years	229 (39.1)	74 (38.3)	155 (39.4)	
≥ 12 years	222 (37.9)	69 (35.9)	153 (39.0)	
Income in MW*				0.914
0–2	91 (16.3)	31 (16.1)	60 (15.3)	
2.1–5	202 (36.3)	64 (33.2)	138 (35.1)	
> 5.1	264 (47.4)	87 (45.1)	177 (45.0)	
Dependence AVD				0.230
No	476 (81.2)	153 (79.3)	323 (82.2)	
Yes	110 (18.8)	40 (20.7)	70 (17.8)	
Fear of falling at baseline				0.522
No	289 (49.3)	95 (49.2)	194 (49.4)	
Yes	297 (50.7)	98 (50.7)	199 (50.6)	

*MW = minimum wage in 2008 ($415.00)

18 missing data

Source: Frailty in Older Adults (FIBRA-RJ) - 2008–2013.

Table 1 shows that 72% of the participants were women, 47.1% from 65 to 74 years old, 44% married or living with a companion, 78.5% had more than six years of schooling and 45% earned more than five minimum wages.

The incidence of FOF was 33.5% and persistence was 71.3% within four years. Table 2 shows that, for the incident FOF, being a woman and age were statistically significant, and for persistent FOF, being a woman, age and marital status. These variables were selected as covariates for the multivariate models.

**Table 2:** Incidence and persistence of fear of falling (FOF) in the follow up according to individuals’ socioeconomic and demographic characteristics. FIBRA-RJ Study, Rio de Janeiro, RJ, Brazil.

Characteristics of the sample	Incidence N (%)	p-value	Persistence %	p
Sex				
Male	18 (33.3)		23 (62.1)	
Female	47 (61.8)	0.059	119 (73.4)	< 0.001
Age (years)				
65–69	8 (19.0)		32 (94.1)	
70–74	26 (63.4)		35 (73)	
75–79	15 (48.4)		31 (72)	
80–84	10 (90.9)		29 (59.1)	
≥ 85	6 (83.3)	0.019	15 (60)	0.006
Marital status				
Married or with companion	29 (39.7)		49 (69.0)	
Divorced/Separated	6 (100.0)		17 (94.4)	
Single	7 (38.9)		20 (83.3)	
Widowed	23 (69.7)	0.218	56 (65.1)	0.001
Schooling				
Illiterate	1 (100.0)		0 (0.0)	
1–4 years	11 (91.7)		24 (77.4)	
≥ 5 years	53 (45.3)	0.249	118 (71.1)	0.106
Income in MW*				
0–2	6 (37.5)		17 (54.8)	
2.1–5	29 (61.7)		43 (61.4)	
> 5.1	28 (46.6)	0.154	78 (87.6)	0.428

*MW = minimum wage in 2008 ($415.00)

Source: Frailty in Older Adults (FIBRA-RJ) - 2008–2013.

The adjusted relative risk showed that “using seven or more medicines” (RR = 2.0; 95%CI: 1.08–3.65) and “reporting worse activity level than at baseline” (RR = 2.0; 95%CI: 1.40–3.07) doubled the risk of occurrence of fear of falling, whereas reduced hand grip strength showed a borderline association in the final model (RR = 1.64; 95%CI: 0.98–2.77) (model 2), as shown in Table 3.

**Table 3:** Crude and adjusted relative risks (RR) and respective 95% confidence intervals (95%CI) for the association between incident fear of falling and clinical/functional, psychosocial and lifestyle variables – FIBRA-RJ Study, Rio de Janeiro, RJ, 2010. (N=65).

Incident fear of falling	Crude RR (95%CI)	Adjusted RR (95%CI) Model 1	Adjusted RR (95%CI) Model 2
Number of morbidities			
0–1	1	1	1
2–3	0.90 (0.58–1.39)	0.84 (0.55–1.29)	0.60 (0.37–0.95)
≥ 4	1.93 (1.13–3.26)	1.75 (1.01–3.04)	0.99 (0.49–1.98)
Number of medicines			
0–3	1	1	1
4–6	1.45 (0.93–2.27)	1.35 (0.87–2.10)	1.40 (0.88–2.24)
≥ 7	2.35 (1.44–3.83)	2.18 (1.36–3.47)	2.00 (1.08–3.69)
Falls			
0	1	1	
1–2	0.82 (0.39–1.66)	0.71 (0.35–1.44)	0.67 (0.36–1.37)
≥ 3	1.98 (0.87–4.54)	1.37 (0.72–2.58)	1.44 (0.55–3.75)
Fracture after fall			
No	1	1	1
Yes	1.50 (0.37–6.12)	1.08 (0.26–4.44)	0.83 (0.25–2.73)
Walking aid			
No	1	1	1
Yes	1.20 (0.40–3.59)	1.40 (0.44–4.42)	1.04 (0.44–2.47)
Hearing impairment			
No	1	1	1
Yes	1.32 (0.72–2.40)	1.37 (0.77–2.44)	1.48 (0.83–2.65)
Visual impairment			
No	1	1	1
Yes	0.85 (0.55–1.32)	0.87 (0.57–1.31)	1.20 (0.73–1.97)
Hand grip strength			
Normal	1	1	1
Impaired	1.77 (1.13–2.79)	1.29 (0.76–2.18)	1.64 (0.98–2.77)
Walking speed			
Normal	1	1	1
Impaired	1.33 (0.77–2.26)	1.00 (0.59–1.70)	1.12 (0.65–1.94)
Functional dependence (BADL)			
No	1	1	1
Yes	1.27 (0.53–3.01)	1.28 (0.59–2.77)	1.59 (0.80–3.13)
Functional dependence (IADL)			
No	1	1	1
Yes	1.48 (1.01–2.19)	1.41 (0.97–2.08)	1.13 (0.73–1.77)
Self-rated health			
Good/Very good	1	1	1
Fair	1.49 (1.00–2.22)	1.51 (1.03–2.21)	1.38 (0.86–2.20)
Poor/Very poor	1.73 (0.43–7.10)	2.26 (0.53–9.57)	3.36 (0.64–17.6)
BMI			
Eutrophic	1	1	1
Undernourished	1.15 (0.42–3.18)	1.17 (0.42–3.24)	0.99 (0.37–2.66)
Overweight/Obese	1.31 (0.48–3.57)	1.33 (0.48–3.60)	1.33 (0.48–3.65)
Cognitive function			
Normal	1	1	1
Impaired	1.04 (0.57–1.92)	1.34 (0.73–2.47)	1.31 (0.72–2.40)
Depressive symptoms			
Normal	1	1	1
Impaired	1.56 (0.87–2.79)	1.42 (0.80–2.54)	1.46 (0.75–2.85)
Living alone			
No	1	1	1
Yes	1.03 (0.64–1.67)	1.13 (0.69–1.85)	0.89 (0.54–1.48)
Activity level			
Better/Same	1	1	1
Worse	2.52 (1.80–3.62)	2.32 (1.61–3.33)	2.07 (1.40–3.07)

BMI: body mass index; BADL: basic activities of daily living; IADL: instrumental activities of daily living.

Model 1: adjusted for sex and age.

Model 2: adjusted for all variables in model, including sex and age.

Source: Frailty in Older Adults (FIBRA-RJ) - 2008–2013.

Table 4 shows association of persistence of FOF with “using seven or more medicines” (RR = 1.48; 95%CI: 1.10–1.98), a “history of one or two falls” (RR = 1.27; 95%CI: 1.01–1.63), “hearing impairment” (RR = 1.36; 95%CI: 1.09–1.71), “diminished walking speed” (RR = 1.36; 95%CI: 1.05–1.48), “poor/very poor self-rated health” (RR = 1.43; 95%CI: 1.13–1.82), “cognitive impairment” (RR = 1.58; 95%CI: 1.15–2.18) and “depressive symptoms” (RR = 1.50; 95%CI: 1.08–2.08) in the final model (model 2).

**Table 4:** Crude and adjusted relative risks (RR) and respective 95% confidence intervals (95%CI) for the association between persistent fear of falling and clinical/functional, psychosocial and lifestyle variables – FIBRA-RJ Study, Rio de Janeiro, RJ, 2010. (N=328).

Persistent fear of falling	Crude RR (95%CI)	Adjusted RR (95%CI) Model 1	Adjusted RR (95%CI) Model 2
Morbidities			
0–1	1	1	1
2–3	1.36 (1.04–1.77)	1.20 (0.93–1.56)	0.90 (0.70–1.20)
≥ 7	2.00 (1.50–2.67)	1.65 (1.24–2.20)	0.92 (0.64–1.35)
Number of medicines			
0–3	1	1	1
4–6	1.42 (1.06–1.90)	1.25 (0.94–1.67)	1.05 (0.79–1.40)
≥ 7	2.24 (1.72–2.90)	1.90 (1.46–2.47)	1.48 (1.10–1.98)
Falls			
0	1	1	1
1–2	1.52 (1.21–1.92)	1.39 (1.11–1.75)	1.27 (1.01–1.63)
≥ 3	1.99 (1.56–2.52)	1.58 (1.23–2.02)	0.98 (0.71–1.35)
Fracture after falling			
No	1	1	1
Yes	1.71 (1.28–2.28)	1.54 (1.02–2.33)	1.09 (0.72–1.66)
Walking aid			
No	1	1	1
Yes	1.61 (1.23–2.10)	1.35 (1.02–1.80)	1.08 (0.76–1.54)
Hearing impairment			
No	1	1	1
Yes	1.37 (1.10–1.71)	1.27 (1.02–1.59)	1.36 (1.09–1.71)
Visual impairment			
No	1	1	1
Yes	1.24 (0.99–1.56)	1.13 (0.91–1.40)	1.12 (0.90–1.40)
Hand grip strength			
Normal	1	1	1
Impaired	1.57 (1.26–1.93)	1.39 (1.09–1.76)	1.23 (0.97–1.57)
Walking speed			
Normal	1	1	1
Impaired	1.73 (1.41–2.11)	1.47 (1.19–1.82)	1.36 (1.05–1.48)
Functional dependence (BADL)			
No	1	1	1
Yes	1.60 (1.29–1.98)	1.50 (1.21–1.84)	1.00 (0.80–1.29)
Functional dependence (IADL)			
No	1	1	1
Yes	1.47 (1.17–1.85)	1.39 (1.10–1.74)	1.13 (0.90–1.43)
Self-rated health			
Good/Very good	1	1	1
Fair	1.64 (1.31–2.06)	1.54 (1.24–1.92)	1.27 (0.73–2.22)
Poor/Very poor	2.08 (1.46–2.97)	1.78 (1.25–2.51)	1.43 (1.13–1.82)
BMI			
Eutrophic	1	1	1
Undernourished	1.01 (0.57–1.80)	1.00 (0.58–1.72)	1.08 (0.62–1.89)
Overweight/Obese	1.22 (0.70–2.13)	1.20 (0.70–2.05)	1.20 (0.70–2.07)
Cognitive function			
Normal	1	1	1
Impaired	1.12 (0.76–1.66)	1.40 (0.97–2.01)	1.58 (1.15–2.18)
Depressive symptoms			
Normal	1	1	1
Impaired	1.59 (1.07–2.34)	1.53 (1.07–2.20)	1.50 (1.08–2.08)
Living alone			
No	1	1	1
Yes	1.29 (1.02–1.62)	1.08 (0.86– 1.37)	1.06 (0.84–1.33)
Activity level			
Better/Same	1	1	1
Worse	1.77 (1.44–2.17)	1.60 (1.31–1.96)	1.17 (0.94–1.46)

BMI: body mass index; BADL: basic activities of daily living; IADL: instrumental activities of daily living

Model 1: adjusted for sex, age and marital status

Model 2: adjusted for all variables in model, including sex, age and marital status

Source: Frailty in Older Adults (FIBRA-RJ) - 2008–2013.

## DISCUSSION

Our study showed that older adults using seven or more medicines and with an worse activity level than the prior year presented a two-fold increased risk of incidence of FOF than those without these conditions. Among those who already presented FOF at the baseline, their risk of continuing to have that fear at the four-year follow-up was greater for those using seven or more medicines, having a history of one or two falls in the prior year, a hearing impairment, diminished walking speed, poor/very poor self-rated health, cognitive impairment and depressive symptoms.

The 33.5% incidence of FOF in our study is consistent with the findings of other international and longitudinal studies, which range from 13.7% to 45.4%^7^. To the best of our knowledge, no longitudinal studies with FOF as outcome have been conducted in Brazil. These findings vary rather widely possibly due to the use of a simple dichotomous question to assess FOF, whereas our study evaluated it using a more specific instrument, the FES-I-BR, which may have reduced the number of false negatives. Although our article sought to evaluate the FOF through a self-efficacy assessment tool (FES-I), thus containing questions related to the individual’s concern about falling, a comparison with the few longitudinal studies on the subject is difficult, since, with the exception of one study^15^, they also assessed FOF using a simple question. Moreover, cultural differences may influence older adults’ perception of their own health status. Clemson et al.^11^ found that not being Australian, European or American was a risk factor for the incidence of fear of falling, which shows the importance of evaluating FOF in different contexts and localities.

Few studies that have evaluated the FOF persistence have found percentages ranging from 46% to 80%^7,9^, being similar to our results, which found 71.3%. It has proven the importance of evaluating FOF persistence, since these other authors have shown substantial differences in the predictors for incident and persistent FOF.

Among risk factors for the incidence, we found that using seven or more medicines and reporting worse activity level than in the prior year increased the risk of developing fear of falling. We emphasize that both factors are modifiable and susceptible to intervention. Friedman et al. (2002)^12^ found that the use of four or more medicines (considered polypharmacy in older adults) was a risk factor. Austin et al.^7^ found that using medicines for depression and/or anxiety was also a risk factor. Our study only evaluated the associations of medicines by the total number in use, regardless of their specific classes.

In our study, the number of morbidities showed no association with FOF, a finding different of that observed by Dierkinger et al.^10^. Our findings must be carefully interpreted, since morbidity was self-reported and older adults tend to over-report morbidities independently of FOF, which could have underestimated the association. Furthermore, our findings alert that polymedication and drug interaction among older adults may be even more important than the number of diseases itself.

Reporting worse activity level than in the prior year was associated with FOF incidence, showing that activities may have not been restricted due to the FOF, but actually before its appearance^10^. This antecipated restriction can be explained by the fact that reduced activity is also related to loss of physical condition, impaired balance and gait and social isolation, thus favoring FOF emergence.

Another study^10^ that analyzed the relationship between reduced social participation and FOF found that a greater interaction with friends could prevent older adults from developing fear of falling. On the other hand, a greater interaction with the family was considered a risk factor. That is, rehabilitation programs can encourage older adults to socially participate and engage them in general activities aiming at preventing them from reducing their activity level.

Reduced hand grip strength indicated a high relative risk of incident fear of falling. This measurement is also considered to be an item in frailty evaluation and can represent the older adult’s physical capacity. No longitudinal studies identifying a relationship between these variables were found.

Other risk factors were associated with persistent FOF. The use of seven or more medicines was also a risk factor for persistence. Studies have assessed persistence by the use of psychotropics or medicines for depression and anxiety^7^, disregarding the total number of medicines.

A history of one or two falls in the prior year was found to be a risk factor for the persistence, which does not increase with the number of falls. Although this association is well established in cross-sectional studies^3,26^, a history of one or more falls entails risk of persistence; however, when three or more falls occur, other factors such as restricted activities and loss of physical condition may be more important. Oh-Park et al.^9^ found that a report of any fall in the prior year was a risk factor for persistent FOF.

Hearing impairment was found to be a risk factor for FOF persistence, which may be associated with balance-related vestibular alterations and with the fact that an impaired hearing may cause some social isolation by hindering communication and social interaction. In our study, this variable was self-reported by answering a question on hearing difficulty. Reporting visual impairment, which is well established as a risk factor for falls, showed no association with FOF persistence. Although some studies have considered balance disorders or dizziness, no studies evaluating this association were found^8,12^.

Considering the multivariate model 2, an impaired walking speed increased the risk of persistent FOF. Oh-Park^9^ showed similar findings, despite the different evaluation, which used an instrument that classified clinically-observed gait as normal or impaired. Another study evaluated gait and dynamic balance by the Timed Up and Go test (TUG), also finding an association^11^. Gait, which represents the older adult’s degree of functionality, has shown to be extremely important to evaluate fear of falling. Our study also evaluated the use of a walking aid, which is an indicator of impaired gait, without finding any association with FOF; differently from a study by Austin et al.^7^, which could find an association of this variable with FOF.

Poor/very poor self-rated health status was also a risk factor for FOF persistence. This variable can be considered a proxy for the older adult’s real health status, being also related to physical and functional decline.

Cognitive impairment and depressive symptoms were found to be risk factors for FOF persistence. Clemson et al.^11^ also found an association between cognitive impairment – although assessed by another instrument, the Organic Brain Syndrome Scale – and FOF occurrence in a five-year average follow-up study. That study did not evaluate the persistence of the fear. The findings in the literature are inconsistent, considering that another study found that a higher MMSE score was a risk factor for FOF occurrence^10^. The authors explained this finding by the fact that these individuals were probably more accurate when answering about the existence of fear. Future investigations should better evaluate this relationship.

Our results pointed out depressive symptoms as a risk factor for persistence of FOF, being consistent with other studies which have shown that they may be a long-term consequence of FOF rather than a risk factor for its occurrence^9^. Moreover, a study with 1,282 female older adults found that using medicine for anxiety or depression was a risk factor for FOF persistence^7^. Another study, on the other hand, found no association with the use of psychotropic medicine^9^. In fact, in our study, older adults with depressive symptoms were 50% more likely to have FOF when compared with those without depressive symptoms.

The limitations of our study include the small sample size and limited representativeness, since our sample was composed by older adults with health insurance and thus with better access to health services and higher socioeconomic position than Brazilian older adults in general population. Assessment in only two stages may have not managed to gauge the stability of FOF over time^15^. Future studies could include further stages over time and at shorter intervals. There were losses at the follow-up, but the analyses showed that these individuals were no different from the study participants regarding their socioeconomic and demographic characteristics, existence of fear of falling at baseline and dependence in ADL. However, this drop-out rate could have influenced the size of the sample and resulted in some imprecision, as well as potential follow-up bias. Some unexplored factors, such as those related to household and external environment, including accessibility, anxiety and the existence of social support, should be included in future longitudinal studies on FOF.

Our study strength points are, first the measurement of FOF performed using the FES-I-BR, an instrument specifically validated to this purpose, and therefore, less likely to include false negatives, thus assuring a more robust concept of FOF, which is relatively subjective and does not constitute a diagnosis. It can thus be inferred that the associations found are also more robust. Second, the study population comprised men and women aged 65 years or more, resulting in a broader sample. Third, our study also included variables in both physical and functional dimensions, as well as psychosocial and lifestyle variables. Finally, the longitudinal design yields more knowledge of the role of risk factors in the occurrence and persistence of fear of falling, being our study the first study of this nature to be conducted in Latin America.

In conclusion, our study showed a high FOF incidence and persistence among older adults after 4 years, which shows that the FOF is an important condition in their lives and should always be evaluated within a multidimensional assessment. This condition is related to a worse quality of life, deserving attention in the field of public health and gerontology, and not only the falls, which are already better established in the literature. Through knowledge of risk factors for both the incidence and persistence of FOF, effective prevention strategies can be established for this population in health services addressing issues other than the prevention of falls.
